# Body size, maturation and motor performance in young soccer players: relationship of technical actions in small-sided games

**DOI:** 10.5114/biolsport.2023.110749

**Published:** 2022-01-03

**Authors:** Julio Cesar da Costa, Paulo Henrique Borges, Luiz Fernando Ramos-Silva, Vinicius Muller Reis Weber, Alexandre Moreira, Enio Ricardo Vaz Ronque

**Affiliations:** 1 Laboratory of Physcial Activity and Health, Center of Physical Education and Sports, Londrina State University – UEL, Londrina, Paraná, Brazil; 2 Department of Physical Education, School of Sports, Universidade Federal de Santa Catarina, Florianópolis, Santa Catariana, Brazil; 3 Physical Education and Sport School, Sports Department, University of São Paulo, São Paulo, São Paulo, Brazil

**Keywords:** Athletic performance, Soccer, Youth, Growth, Maturity

## Abstract

The objective of this study was to investigate the relative contributions of body size, skeletal age, and motor performance variables with technical actions through an ecological model during small-sided soccer games, and the interaction of biological maturation with technical and motor performance in young players. In this cross-sectional study, eighty-two young players (14.4 ± 1.1 years), belonging to state-level soccer teams and divided by category (U-13 and U-15), were included. Players having an injury in the evaluation period were not included in the study. Measurements of body size, skeletal age (SA), motor tests, and technical actions in small-sided games (SSG) were performed (3 × 3 plus goalkeeper) in two periods (halves) of four minutes. Differences between age groups were found for SA (ES = -2.36), chronological age (ES = -3.89), body mass (ES = -2.09), height (ES = -1.90), and fat-free mass (ES = -2.09). Positive associations were found between body size (R = 0.43 to R = 0.48) and manipulation (R = 0.50 to R = 0.52) indicators and numbers of technical actions (CB and SS), except for stature with LB (R = -0.42) in the U-13 age group. In the U-15 category, skeletal age (R = -0.29 to R = -0.30) and body mass (R = -0.28 to R = -0.29) were negatively associated with the number of technical actions (RB, NB, LB, and OB) (P > 0.05) and positively with the balance with LB (R = 0.26). In conclusion, body size, SA, and motor performance influenced technical actions in SSG differentially in each category. U-13 heavier players and those with a better motor performance presented higher involvement due to the higher

## INTRODUCTION

Adolescence is marked by great variability, especially in body size characteristics. This variability in adolescence is notorious at the initial stage of the long-term training process of young soccer players [1]. Such large differences among young players makes the issue of identifying and developing a talent a complex and very difficult task [2], mainly due to the well-known influence of maturation on the performance indicators of young soccer players [3, 4].

The profile of young soccer players differs from the general population at the same age group. In general, they have greater body size and strength [3–6]. Nevertheless, in practical settings, it is possible to observe the participation of some players with smaller body size, compared to their peers. Indeed, it is not unusual that smaller players and with lower skeletal age present worse performance in motor and specific skill tests, in particular, in tests related to speed and agility attributes [7]. Due to the presence of these inequalities in the sports environment, young soccer player with smaller body size and lower skeletal age tend to be excluded during the long-term training process, mainly due to the influence of biological maturation at this stage of life [5, 6, 8].

However, excluding “smaller” players from the training process can be harmful to the team in the medium term, since at the end of adolescence, the physical advantages promoted by body size differences will no longer be significant [9]. Therefore, evaluating them through isolated soccer-specific skill tests might neglect technical qualities performed in real situations, since these batteries are usually influenced by functional capacities, overestimating advanced players in maturation processes [10], which brings the need for new methods such as the number of technical actions, helping professionals involved in the training and evaluation of young soccer players. In addition, the interaction between maturation and age may provide certain advantages for older soccer players in the selection and training process [11], especially when the biennial category division process is adopted; for example, differences between game categories have been observed when comparing body size, skeletal age, and performance indicators in various batteries of specific skill tests [12, 13].

Although the results of several studies have demonstrated the physical advantages that maturation exerts on sport performance [5, 14], the influence of the body growth process on the number of technical actions performed by young soccer players during a soccer match still remains unclear, given that the majority of studies in this field have been mainly focused on the influence of biological maturation on the performance of isolated skill tests, therefore demonstrating low ecological validity [15], reflecting the absence of complex decision-making processes, and thus neglecting the systemic characteristics of soccer, which is influenced by constraints imposed by the individual, environment and task triad [16]. In this way, the evaluation of the number of technical actions during games, together with body growth, skeletal age and motor performance in an ecological context, can help in assessing the influence of the body growth process on the participation of players in the game, which in turn could be analysed due to a multivariate approach [17], therefore assisting training professionals in monitoring and selecting young soccer players [18].

In this context, small-sided soccer games (SSG) have been considered as training drills with the possibility of assessing technical performance [19], and have been proposed to be a viable tool for evaluation of the number of technical actions [1], contemplating technical, tactical and physical aspects of the soccer game [20], which is thought to promote stimuli similar to those found in official match situations [21].

Despite showing moderate variability in technical actions between training sessions, SSG have also been shown to be a reliable tool for systematic monitoring of technical performance in an ecological model, since they increase individual participation in the number of technical actions [22], mimicking the unpredictive feature of the game, requiring participants to adjust their movements according to individual, environmental and task dynamic constraints [16], and presenting high stability in technical performance across a 16-month training period in elite young soccer players [19].

Observing the performance of young players during SSG and assessing the influence of body growth and maturation on SSG performance could help coaching staff and practitioners working with the training of young soccer players in formulating better guidance that would assist planning, monitoring and developing players based on their size and motor characteristics. Based on previous information, our hypothesis was that there would be differences in the involvement (number of performed technical actions) of young soccer players in SSG due to body size differences and maturational status. Thus, the aim of this study was to evaluate the relative contributions of body size, skeletal age and motor performance variables on technical actions through an ecological model during small-sided soccer games, and the interaction of biological maturation and technical and motor performance in young players.

## MATERIALS AND METHODS

### Participants

Among the ninety-seven eligible young soccer players, the final sample was composed of eighty-five athletes, and their training was composed of 4–5 weekly sessions, with training session duration of 120–180 min, including soccer training sessions, small-sided games, and strength and conditioning training sessions, as previously described [23]. The study was conducted in accordance with the National Health Council resolution (466/2012) and was approved by the Research Ethics Committee of the local university (Proc. 2.650.232/2018). As inclusion criteria, it was required that the players belong to the youth academy team, not being in the pre-evaluation period in the club and not having any kind of injury during the evaluation period. Thus, data from 15 players who did not meet these criteria were removed from the final analysis [23].

### Experimental design

In this cross-sectional study, data collection occurred between September and October 2018, just before the beginning of the teams’ pre-season. Data collection occurred on four days (D1, D2, D3, and D4). On the first day (D1), anthropometric measurements and motor performance tests were conducted; on the second day (D2), body composition measurements were performed using the plethysmography method; on day three (D3), players had their wrists X-rayed; and on the last day (D4), SSG were performed (3 × 3). Athletes were instructed to recover 48 hours before the beginning of data collection and the recovery time was 21 hours between tests. All players were familiar with tests and experimental procedures.

### Anthropometry and body composition

Body mass and height measurements were obtained, according to criteria described by Gordon, Chumlea and Roche [24]. Fat-free mass was estimated by the whole-body plethysmography method using the Body Composition System (BOD POD, Life Measurement Inc., Concord, CA, USA) estimated by body density based on the two-component model, using the specific equation of Siri [25] and adjusted by age-specific constants described by Lohman [26].

### Chronological and skeletal age

To establish the chronological age (CA) in centesimal form, the difference between the date of birth of the player and the date of evaluation of the wrist and left-hand X-ray was calculated. Players were classified by category according to their year of birth; U-13 for those born in 2005 and 2006 and U-15 for those born in 2003 and 2004. Skeletal age was measured through anteroposterior X-ray of the left hand and wrist performed in a specialized laboratory; skeletal age (SA) was estimated by means of the TW3 method [27] by a single trained observer; the method uses individual skeletal age assignment for 13 hand and wrist bones through radiographs. Twenty radiographs (20%) were blindly assessed a second time after 15 days. The intra-class correlation coefficient found was 0.97 (*P* < 0.01) and intra-observer error was 0.26 years.

### Small-sided games (SSG)

SSG in the 3 × 3 plus goalkeeper was used in this study [20, 28]. This format has been used for simulating the demands of the game and developing technical skills [29], in addition to being a tool for identifying/monitoring young talents [30]. SSG consisted of two periods (halves) of four minutes. Passive recovery of 1 min was allowed between halves. The pitch area adopted was 36 m (width) × 27 m (length) [20, 28]. All players were largely familiarized with this SSG format, as it was habitually used in their training routines. Each player participated in only one SSG.

**FIG. 1:**
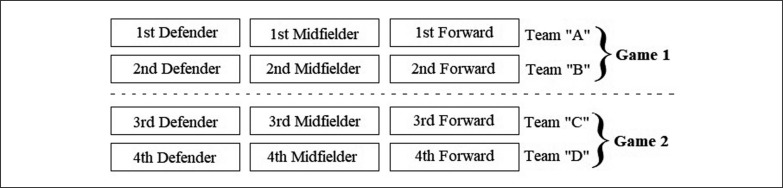
Team composition according to the specific skill results.

Within each age group, teams were arranged based on two criteria: ranking in the specific battery skill results [23, 31, 32], and game position (Figure 1).

In the first confrontation, team A consisted of the best defender, best midfielder and best forward, while team B consisted of the second-best defender, second-best midfielder and second-best forward. This counterbalanced procedure was adopted to allow similar technical performance conditions between teams.

### Technical actions in SSG

The number of technical actions was adopted as an indicator of technical performance [33]. The number of technical actions was recorded during SSG using a camera (Casio EX-10, 30 Hz frequency acquisition, São Paulo - Brazil) located 6 m above and to one side of the pitch long axis at a distance of 15 m from the pitch. The Lince software was used to code SSG technical actions. According to the specific taxonomy from the observational methodology, this study can be classified as nomothetic, punctual and multidimensional [34]. The following technical actions were recorded: conquering the ball (CB), receiving the ball (RB), playing a neutral ball (NB), losing the ball (LB), playing an offensive ball (OB), and successful goal attempts (SS). The procedures adopted for each of the technical actions were described in a previous study [23]. This tool has validity of 0.74 [35].

Twenty young soccer players (20%) were blindly re-evaluated a second time after 15 days. The intraclass correlation coefficient found for CB was 0.83 (P < 0.01), for RB 0.96 (P < 0.01), for NB 0.97 (P < 0.01), for LB 0.96 (P < 0.01), for OB 0.98 (P < 0.01) and for SS 0.96 (P < 0.01).

### Motor performance

To evaluate motor performance, a battery of tests proposed by Luz et al. [36] was adopted, using six tests to evaluate locomotion, balance, and manipulation. This battery has very good validity for this population (*X*² = 12.04, p = 0.061; NFL = 0.982; CFI = 0.991; RMSEA = 0.059). For locomotion, the shuttle run and long jump tests were applied. In the shuttle run test, the player runs from one line to another separated by 10 m and picks up two wooden blocks, one at a time; when running back, the athlete must place the block beyond the starting line. The final result of the test was the time spent on the course; the test was applied twice and only the best time obtained was considered. In the long jump, the athlete jumped from one line with both feet simultaneously. The player repeated the test twice, and the greatest distance (in metres) between the start line and the back of the heel on landing closest to the start line was used as the final result. To evaluate balance, the platform displacement and lateral jump tests were performed. On the lateral displacement platform, the player walked laterally using two wooden platforms (25 cm × 25 cm × 2 cm) for 20 seconds. For each successful transfer between platforms, two points were scored, one for each platform; the test was applied twice and only the best result was used. In the side jump, the athlete jumped laterally with both feet together over a five cm beam as fast as possible for 15 seconds. For each successful jump, one point was scored; the test was applied twice and only the best result was used. For manipulation, throwing speed and kicking speed tests were performed. In the throwing speed test, the player throws a baseball at maximum speed against a wall. Two attempts were made, and the final result was the highest speed throw. For the kick speed, kicks were performed at maximum speed against a wall with the player’s preferred leg. Two attempts were made and the final result was the kick with the highest speed. Subjects were familiarized with tests through an attempt prior to evaluation. 2D analysis, kicks, and throws were recorded by a camera, model Casio EX-10 (Cassio, São Paulo, Brazil), at an adjusted rate of 240 Hz and laterally positioned at a distance of 5 m from the central test area to allow observation of the ball trajectory. The area where subjects performed the tasks was calibrated using four metal rods, in an area of 2.0 × 1.6 m^2^, with eight known Cartesian coordinate points [37].

For calculation of the throwing and kicking speed, 2D images were reconstructed using the Dvideo Software [38]. The first ten frames after the last favourite hand or foot lost contact with the ball were used for reconstruction. 2D distances covered by the ball were calculated in the Microsoft Excel software and estimated through calculation of the Euclidean distance and distance in metres per second (m/s).

The results of the six tests were transformed into a Z score for further analysis. To obtain the locomotion variable, the results of the shuttle run and long jump tests were added; for the balance variable, the results of the platform displacement test and lateral jump test were added; and for the manipulation variable, the results of the throwing speed and kicking speed tests were used. Twenty young soccer players (20%) were blindly re-evaluated a second time after 15 days. The intraclass correlation coefficient found for the shuttle run test was 0.84 (P < 0.01), for the long jump test, 0.93 (P < 0.01), for the platform displacement test, 0.78 (P < 0.01), for the lateral jump test, 0.94 (P < 0.01), for the throwing speed test, 0.92 (P < 0.01) and for the kicking speed test, 0.95 (P < 0.01).

### Statistical analysis

Data are described as mean and 95% confidence interval. Data normality was evaluated by the asymmetry and kurtosis graph method, and variables that did not present normality were fitted by a logarithmic equation. The independent t test was used to compare U-13 and U-15 categories for body size, skeletal age, motor performance and technical actions and the effect size was observed by Hedges’ g. Pearson’s correlation coefficient was applied to evaluate the relationship between body size, skeletal age, and motor performance and technical actions by age group and only variables that showed p < 0.05 were added to the linear regression model. Finally, linear regression was adopted to assess the power of explanation between the variables motor performance, body size and skeletal age, and those of technical actions adjusted by motor performance, body size and skeletal age. The significance adopted was 5% and the software used was SPSS 24.0 (IBM Corp., Armonk, NY).

## RESULTS

Table 1 describes the sample characteristics stratified by category (U-13 and U15), for technical actions, anthropometric and motor performance variables. Players had a mean chronological age of 14.4 ± 1.1 years. When comparing age groups, significant differences were found for all anthropometric variables. A higher SA value was observed for U-15 soccer players (ES = -2.36), who also had greater chronological age (ES = -3.89), body mass (ES = -2.09), height (ES = -1.90), and fat-free mass (ES = -2.09). Technical actions and motor performance variables did not show differences between categories.

**TABLE 1:** Body size, skeletal age, motor performance and SSG technical actions by category (mean and CI 95%)

Variables	Total Sample	U-13 (n = 24)	U-15 (n = 58)	*t*	*P*
Chronological age (years)	14.4 (14.1–14.6)	12.9 (12.6–13.1)	15.0 (14.9–15.1)	-16.191	< 0.001*
Skeletal age (years)^#^	14.7 (14.7–14.3)	13.0 (12.4–14.5)	15.4 (15.1–16.0)	-9.753	< 0.001*
Body mass (kg)	57.9 (55.6–60.2)	46.8 (42.9–50.6)	62.5 (60.7–64.2)	-8.698	< 0.001*
Stature (cm)	169.5 (167.3–171.8)	159.4 (155.1–163.6)	173.8 (172.1–175.5)	-7.884	< 0.001*
Fat free mass (kg)	51.7 (48.5–53.9)	41.0 (37.0–45.0)	56.2 (54.6–57.8)	-8.694	< 0.001*
Locomotion (score)	0.0 (-0.1–0.1)	0.0 (-0.2–0.2)	0.0 (-0.1–0.1)	-0.169	0.867
Balance (score)	0.0 (-0.2–0.2)	0.0 (-0.4–0.4)	0.0 (-0.2–0.2)	-0.002	0.999
Manipulation (score)	0.0 (-0.2–0.2)	0.0 (-0.3–0.3)	0.0 (-0.2–0.2)	-0.036	0.972
Conquering the ball (number of actions)^#^	1.9 (1.5–2.2)	2.1 (1.5–2.7)	1.8 (1.4–2.2)	0.657	0.513
Receiving the ball (number of actions)	10.6 (9.9–11.4)	10.9 (9.5–12.3)	10.5 (9.6–2.2)	0.421	0.675
Neutral ball (number of actions)	6.7 (6.1–7.4)	6.7 (5.4–8.0)	6.7 (6.0–7.6)	-0.068	0.946
Losing the ball (number of actions)	2.6 (2.3–3.0)	2.4 (1.8–3.2)	2.7 (2.3–3.2)	-0.630	0.530
Playing an offensive ball (number of actions)	1.9 (1.6–2.5)	1.6 (1.1–2.1)	2.0 (1.6–2.5)	-1.031	0.306
Executing a successful shot (number of actions)^#^	2.1 (1.7–2.4)	1.7 (1.1–2.3)	2.2 (1.8–2.7)	-0.543	0.591

Note: # = transformed data log^10;^

* = significant difference between U-13 and U-15 category (P < 0.05).

Table 2 presents correlations between body size, skeletal age and motor performance and number of technical actions stratified by category. For the U-13 category, the number of technical actions showed weak to moderate positive correlations with body size and motor performance indicators (*P* < 0.05). Body size (body mass and fat-free mass) presented positive correlations with CB and SS. In addition, manipulation presented positive correlations with CB and SS. Regarding the number of technical actions, smaller soccer players presented a higher number of LB.

**TABLE 2:** Correlation between body size, skeletal age and motor performance variables with the SSG technical actions by category

Variables	Conquering the ball^#^	Receiving the ball	Neutral ball	Losing the ball	Playing an offensive ball	Executing a successful shot^#^
	r (CI 95%)	r (CI 95%)	r (CI 95%)	r (CI 95%)	r (CI 95%)	r (CI 95%)
**U-13 (n = 24)**						

Skeletal age (years)^#^	0.37 (0.05–0.67)	0.08 (-0.32–0.48)	-0.10 (-0.48–0.32)	-0.32 (-0.63–0.11)	0.28 (-0.12–0.62)	0.34 (-0.13–0.69)
Body mass (kg)	0.48 (0.09–0.74)*	0.09 (-0.32–0.48)	-0.06 (-0.45–0.35)	-0.34 (-0.65–0.08)	0.27 (-0.15–0.61)	0.46 (0.06–0.73)*
Stature (cm)	0.33 (-0.07–0.65)	0.06 (-0.35–0.45)	-0.07 (-0.46–0.34)	-0.42 (-0.70 – -0.02)*	0.17 (-0.25–0.53)	0.48 (0.10–0.74)*
Fat free mass (kg)	0.45 (0.05–0.71)*	-0.06 (-0.45–0.35)	-0.20 (-0.55–0.22)	-0.32 (-0.63–0.10)	0.18 (-0.24–0.54)	0.43 (0.03–0.71)*
Locomotion (score)	0.12 (-0.31–0.48)	0.32 (-031–0.49)	-0.41 (-0.42–0.38)	0.41 (-0.28–0.51)	-0.34 (-0.44–0.36)	0.13 (-0.63–0.76)
Balance (score)	0.34 (-0.07–0.66)	-0.06 (-0.45–0.35)	0.10 (-0.32–0.48)	-0.16 (-0.53–0.26)	-0.23 (-0.58–0.18)	0.03 (-0.38–0.42)
Manipulation (score)	0.52 (0.15–0.76)*	0.31 (-0.11–0.63)	0.14 (-0.28–0.51)	-0.23 (-0.59–0.19)	0.30 (-0.12–0.63)	0.50 (0.12–0.75)*

**U-15 (n = 58)**						

Skeletal age (years)^#^	0.12 (-0.16–0.37)	-0.30 (-0.52– -0.05)*	-0.29 (-0.50– -0.02)*	-0.09 (-0.32–0.19)	0.19 (-0.41–0.08)	-0.18 (-0.46–0.02)
Body mass (kg)	-0.06 (-0.31– 0.20)	-0.25 (-0.48–0.01)	-0.29 (-0.51– -0.03)*	-0.03 (-0.29–0.23)	-0.28 (-0.50–0.02)*	-0.17 (-0.41–0.09)
Stature (cm)	-0.10 (-0.34–0.16)	-0.22 (-0.46–0.04)	-0.22 (-0.45–0.04)	0.09 (-0.34–0.17)	-0.23 (-0.46–0.03)	-0.17 (-0.40–0.10)
Fat free mass (kg)	-0.06 (-0.31–0.20)	-0.14 (-0.39–012)	-0.20 (-0.44–0.06)	-0.14 (-0.36–0.15)	-0.22 (-0.45–0.04)	-0.17 (-0.39–0.11)
Locomotion (score)	-0.14 (-0.50–0.25)	-0.04 (-0.40–0.32)	-0.07 (-0.41–0.30)	-0.16 (-0.49–0.21)	-0.42 (-0.68–0.07)	-0.27 (-0.59–0.11)
Balance (score)	0.15 (-0.11–0.39)	0.17 (-0.09–0.41)	0.07 (-0.19–0.32)	0.26 (0.01–0.49)*	-0.05 (-0.30–0.21)	0.01 (-0.25–0.26)
Manipulation (score)	-0.13 (-0.39–0.12)	0.23 (-0.03–0.45)	0.04 (-0.22–0.29)	-0.04 (-0.29–0.22)	-0.08 (-0.33–0.18)	0.01 (-0.20–0.31)

Note: # = transformed data log^10^;

* = significant correlations (P < 0.05).

In the U-15 category, players with lower weight or lower skeletal age showed weak inverse correlations with number of technical actions (*P* < 0.05); SA was significantly correlated with RB and NB, and body mass showed significant correlations with NB and OB. In addition, balance was positively correlated with LB.

The association between body size, skeletal age, motor performance and number of technical actions in SSG is described in Table 3. Regression models were applied only to technical variables that presented significant correlations with body size and motor performance in the U-13 category. The analysis revealed that the number of technical actions CB and SS was positively associated with body size indicators (*P* < 0.05). However, when using the adjusted model (models 2 and 3), associations did not present significance. In motor performance, it was observed that for each extra unit in the manipulation test, the number of technical actions increased by 0.52 in CB and 0.50 in SS. Although no significant associations were found when motor performance was adjusted for skeletal age (model 3) and body size indicators (model 4) for SS, for CB, the association maintained its significance (*P* = 0.03), demonstrating that this association was not affected by body size variables.

**TABLE 3:** Linear regression between body size, skeletal age, motor performance and SSG technical actions for U-13 (n = 24) category.

Independent variables	Adjustment variables	Linear regression								
		Conquering the ball^#^			Losing the ball			Executing a successful shot^#^		
		β	R^2^	*P*	β	R^2^	*P*	β	R^2^	*P*
**Body Mass**	Model 1	0.48	0.23	0.02*	–	–	–	0.46	0.21	0.02*
	Model 2	0.30	0.34	0.15	–	–	–	0.29	0.31	0.17
	Model 3	0.33	0.23	0.34	–	–	–	0.26	0.22	0.46

**Stature**	Model 1	–	–	–	-0.42	0.17	0.04*	0.48	0.23	0.02*
	Model 2	–	–	–	-0.40	0.18	0.09	0.32	0.33	0.13
	Model 3	–	–	–	-0.51	0.18	0.14	0.34	0.24	0.30

**Fat free mass**	Model 1	0.45	0.20	0.03*	–	–	–	0.43	0.19	0.03*
	Model 2	0.34	0.30	0.13	–	–	–	0.29	0.32	0.14
	Model 3	0.26	0.24	0.32	–	–	–	0.24	0.23	0.36

**Manipulative**	Model 1	0.52	0.27	0.01*	–	–	–	0.50	0.25	0.01*
	Model 3	0.40	0.33	0.61	–	–	–	0.37	0.31	0.08
	Model 4	0.45	0.44	0.03*	_–_	_–_	_–_	0.36	0.33	0.11

Note: Model 1 = Gross Value; Model 2 = Adjusted for motor performance (manipulation); Model 3 = Adjusted for skeletal age; Model 4 = Adjusted for body size variables (body mass, stature and fat free mass);

* = significant correlations (P < 0.05).

Table 4 shows the association between body size, skeletal age, motor performance and number of technical actions in SSG in the U-15 category. The results show negative associations between SA and number of technical actions (RB and NB) and body size with number of technical actions (NB and OB) (*P* < 0.05). For motor performance, positive associations were observed between balance and LB. Associations between SA or body size and number of technical actions were not affected by motor performance (balance), while associations of SA were affected by body size variables and body mass by SA. Regarding associations between balance and LB, the influence of body size and SA was not significant.

**TABLE 4:** Linear regression between body size, skeletal age, motor performance and SSG technical actions for U-15 (n = 58) category

Independent variables	Adjustment variables	Linear regression											
		Receiving the ball			Neutral ball			Losing the ball			Playing an offensive ball		
		β	R^2^	*P*	β	R^2^	*P*	β	R^2^	*P*	β	R^2^	*P*
**Skeletal age^#^**	Model 1	-0.31	0.09	0.02*	-0.29	0.08	0.03*	–	–	–	–	–	–
	Model 2	-0.31	0.12	0.02*	-0.28	0.08	0.03*	–	–	–	–	–	–
	Model 4	-0.26	0.13	0.16	-0.17	0.11	0.40	–	–	–	–	–	–

**Body Mass**	Model 1	–	–	–	-0.29	-0.08	0.03*	–	–	–	-0.28	-0.08	0.03*
	Model 2	–	–	–	-0.31	0.10	0.02*	–	–	–	-0.28	0.08	0.04*
	Model 3	–	–	–	-0.18	0.10	0.31	–	–	–	-0.30	0.08	0.10

**Balance**	Model 1	–	–	–	–	–	–	0.26	0.07	0.05*	–	–	–
	Model 3	–	–	–	–	–	–	0.26	0.08	0.05*	–	–	–
	Model 4	–	–	–	–	–	–	0.32	0.13	0.02*	–	–	–

Note: # = transformed data log^10^; Model 1 = Gross Value; Model 2 = Adjusted for motor performance (Balance); Model 3 = Adjusted for bone age; Model 4 = Adjusted for body size variables (body mass, stature and fat free mass);

* = significant correlations (P < 0.05).

## DISCUSSION

The aim of this study was to verify the impact of body size and motor performance variables on the number of technical actions performed during SSG in young soccer players. The main result of this study was that the number of technical actions in SSG was positively associated with body size and motor performance in the U-13 category, and negatively associated with skeletal age, body size and motor performance in the U-15 category, except for the balance test, which was positively associated with LB technical action.

In our study, which used an ecological model of the game to observe the number of technical actions in SSG, no differences were observed between age groups U-13 and U-15. This fact suggests that the technical elements of the game occurred similarly in different age groups; however, the relative effects of body size on the number of technical actions were still inconclusive using the ecological model in the different age groups.

In the U-13 category, taller, heavier players and those with better performance in the manipulation test showed higher involvement in SSG, with a higher number of defensive (CB) and offensive (SS) actions, whereas shorter players performed a higher number of LB actions. Previous studies have consistently shown that soccer players advanced in biological maturation outperform their peers in strength, height, and body mass [3, 5, 6, 8], but there is still a lack of information about the influence of anthropometric factors [4–6] and maturational factors [39] on the number of technical actions in SSG performed by young soccer players. The present results showed that in the U-13 category, body size influenced the number of technical actions performed during SSG, indicating that taller and heavier players are more prone to present higher participation in SSG (higher involvement) regardless of their skeletal age, which could be a confounding factor, and consequently, higher likelihood of presenting better performance and advantages to be selected for further phases of the training process. Interestingly, this influence was also evident due to the linear regression results, which showed that controlling for factors such as body size and SA, associations between these variables and number of technical actions were not significant.

Contrary to results observed for U-13, in the U-15 category, body size and SA presented an inverse correlation with number of technical actions. In this age category, players with lower body mass and SA presented higher involvement in SSG technical actions (RB, NB, and OB), receiving a higher number of passes, and performing a higher number of both neutral and offensive passes. These findings suggest that in this age category, factors other than anthropometric variables may be involved in performance during SSG, and these factors may be highly associated with technical skills and tactical knowledge of players, regardless of body dimensions.

In a 10-year longitudinal follow-up study, Figueiredo et al. [40] evaluated young soccer players from 11–13 to 22–25 years of age. The authors observed that players who reached professionalization and remained active in soccer were those who presented at the age of 13 years, lower SA, lower body mass, greater ball control and a higher score in the pass test. In addition, those with higher performance in specific skill tests at the age of 15 years reached national level teams. In a study conducted by Craig and Swinton [41], the authors followed 512 young Scottish soccer players aged 10–17 over ten years. The authors observed that successful players have advantage in anthropometric and physical profiles; however, these differences cannot be considered a reliable source for predicting success in professional soccer, highlighting the need for monitoring other characteristics, including technical skills.

Regarding motor performance, U-13 soccer players who showed better motor performance in the manipulation test performed a higher number of technical actions in CB and SS. In U-15, those with better results in the balance tests had higher LB. We expected that the results in the U-15 category would follow correlations obtained in the U-13 category, in which U-13 players presented positive associations between the motor test and technical actions. A possible explanation for this finding in the U-15 category is related to motor tasks, such as dynamic balance, which require certain coordination, strength, and power. In this case, players with advanced SA probably showed better motor task performance. This positive relationship between motor tests and maturity is related to neuromuscular maturation and the recruitment of specific muscle fibres [42]. In addition, the effect of playing position, which was not controlled in this study, may have influenced these associations [43], since mainly U-15 category forwards with better balance are probably more involved in 1 vs 1 situations due to their greater strength and power to determine the outcome, but this hypothesis should be empirically tested in further studies.

Partially contradicting our initial hypothesis, the results of the present study suggest that the influence of body size and maturity indicators become less important in older athletes, and smaller players and those with late maturity present higher involvement in SSG due to the higher number of technical actions performed, especially for those related to offensive actions. However, there is a need to look beyond the current moment of the performance of young soccer players. Body growth and maturity in each category may present different outcomes related to the number of technical actions in a soccer match. The non-exclusion of these players with smaller body size in the U-13 and especially in the U-15 category should be considered because their permanence in the training process could contribute to their long-term development [44], since studies point out that after 17 years of age, late athletes tend not to differ in size and performance from early athletes, demonstrating stabilization [45]. Thus, late athletes could have better athletic potential as adults, due to the various challenges faced in the training process to remain in the group of soccer players and not to be prematurely excluded [46].

As study limitations, we highlight the cross-sectional design, which is unable to follow variations in the performance of young soccer players over time and the low number of late players. In addition, the small sample size due to division by age groups makes it impossible to perform analyses by playing position. Nevertheless, the present study contributes to advancing of the understanding of relationships between the number of technical actions in SSG and body size, maturation and motor performance. The results obtained are novel and unique, add important information to the literature, and provide important clarifications for professionals who work with the selection and development of young soccer players. Therefore, future studies with longitudinal designs are needed, according to changes caused by body size during the growth process in motor performance and technical performance of young players.

## CONCLUSIONS

In conclusion, body size, skeletal age and motor performance can differently influence the number of performed technical actions when U-13 and U-15 categories are compared. In the U-13 category, the positive influence of body size and motor performance indicators shows that heavier players and those with better motor performance are at an advantage in performing a higher number of technical actions CB and SS). In the U-15 category, advantages promoted by physical growth were not observed. Players with lower body mass and SA had a higher number of performed technical actions (RB, NB and OB) during SSG.

## Figures and Tables

**SUPPLEMENTARY TABLE 1:** Normality and homogeneity of motor performance and number of technical actions

Variables	Levene Test	P	Skewness	Std. Error of Skewness	Z Skewness	Kurtosis	Std. Error Of Kurtosis	Z Kurtosis
Locomotion (score)	1.494	0.225	0.147	0.266	0.55	0.740	0.526	1.40
Balance (score)	3.184	0.078	-0.075	0.266	1.40	-0.077	0.526	-0.14
Manipulation (score)	0.125	0.725	0.179	0.266	0.67	-0.416	0.526	-0.79
Conquering the ball (number of actions)^#^	0.257	0.614	0.477	0.276	1.72	-0.516	0.545	-0.94
Receiving the ball (number of actions)	0.702	0.405	0.153	0.266	0.57	-0.516	0.545	-0.94
Neutral ball (number of actions)	0.057	0.812	0.269	0.266	1.01	-0.141	0.526	-0.26
Losing the ball (number of actions)	0.000	0.987	-0.179	0.276	-0.64	-0.969	0.545	-1.77
Playing an offensive ball (number of actions)	3.423	0.068	0.118	0.295	0.40	-0.207	0.582	-0.35
Executing a successful shot (number of actions)^#^	0.007	0.933	0.376	0.281	1.33	-0.736	0.555	-1.32

**Note:** # = transformed data log^10^;

**SUPPLEMENTARY TABLE 2:** Sample description by category and playing position

Variables	U-13 (n = 24)					U-15 (n = 58)				
	Defender (n = 9)	Midfielder (n = 5)	Forward (n = 10)	F	P	Defender (n = 18)	Midfielder (n = 25)	Forward (n = 15)	F	P
Chronological age (years)	12,9 ± 0,5	12,9 ± 0,5	12,9 ± 0,7	0,006	0,994	14,9 ± 0,6	15,0 ± 0,5	15,1 ± 0,5	0,495	0,612
Skeletal age (years)	13,3 ± 1,3	12,4 ± 0,9	12,9 ± 1,5	0,742	0,488	15,4 ± 0,8	15,5 ± 0,7	15,2 ± 1,1	0,420	0,659
Body mass (kg)	50,1 ± 10,5	43,8 ± 6,4	45,3 ± 8,9	0,996	0,386	63,9 ± 5,9	62,3 ± 6,3	61,1 ± 7,8	0,769	0,469
Stature (cm)	162,3 ± 10,8	158,7 ± 7,4	157,1 ± 10,8	0,602	0,557	176,8 ± 7,2	171,9 ± 5,4^a^	173,2 ± 5,4	3,530	**0,036**
Fat free mass (kg)	42,5 ± 12,0	39,9 ± 6,2	40,3 ± 9,0	0,159	0,854	57,5 ± 6,1	55,7 ± 5,4	55,4 ± 7,0	0,632	0,536
Locomotion (score)	-0,05 ± 0,3	0,3 ± 0,6	-0,08 ± 0,4	1,296	0,295	0,09 ± 0,5	-0,01 ± 0,6	-0,1 ± 0,5	0,523	0,596
Balance (score)	-0,15 ± 0,8	0,5 ± 0,6	-0,11 ± 1,02	1,108	0,349	-0,1 ± 0,6	0,0 ± 0,7	0,1 ± 0,7	0,501	0,609
Manipulation (score)	-0,12 ± 0,7	-0,03 ± 0,6	0,13 ± 1,0	0,218	0,806	-0,1 ± 0,9	-0,02 ± 0,07	0,2 ± 1,0	0,583	0,562
CB (n. of actions)^#^	2,0 ± 1,3	2,2 ± 1,3	2,1 ± 1,7	0,029	0,971	1,5 ± 1,1	1,9 ± 1,3	2,1 ± 2,3	0,574	0,567
RB (n. of actions)	9,8 ± 3,7	11,0 ± 1,9	11,9 ± 3,6	0,941	0,406	9,5 ± 3,1	11,0 ± 3,8	11,0 ± 3,4	1,089	0,344
NB (n. of actions)	7,0 ± 2,2	7,8 ± 1,9	5,9 ± 3,9	0,719	0,499	6,4 ± 2,7	7,5 ± 2,8	6,0 ± 3,7	1,309	0,278
LB (n. of actions)	1,8 ± 1,3	1,6 ± 1,1	3,5 ± 1,7^a,b^	4,339	**0,026**	3,0 ± 2,0	2,6 ± 1,5	2,6 ± 1,9	0,311	0,734
OB (n. of actions)	1,5 ± 0,9	1,4 ± 1,3	1,8 ± 1,5	0,192	0,827	1,4 ± 1,1	2,0 ± 1,4	2,3 ± 2,3^a^	3,417	**0,040**
SS (n. of actions)^#^	1,2 ± 1,0	1,8 ± 1,6	2,1 ± 1,8	0,823	0,453	1,8 ± 1,5	2,2 ± 1,4	2,7 ± 2,2	1,366	0,264

**Note**: # = transformed data log^10^; CB = Conquering the ball; RB = Receiving the ball; NB = Neutral ball; LB = Losing the ball; OB = Playing an offensive ball; SS = Executing a successful shot.
